# Effect of Abscisic Acid on Growth, Fatty Acid Profile, and Pigment Composition of the Chlorophyte *Chlorella (Chromochloris) zofingiensis* and Its Co-Culture Microbiome

**DOI:** 10.3390/life13020452

**Published:** 2023-02-06

**Authors:** Tatiana A. Kozlova, Alexander V. Kartashov, Elena Zadneprovskaya, Anastasia Krapivina, Peter Zaytsev, Olga B. Chivkunova, Alexei E. Solovchenko

**Affiliations:** 1 Laboratory of Ecology, Institute of Natural and Technical Systems of RAS, Kurortniy Ave. 99/18, 354024 Sochi, Russia; 2 K.A. Timiryazev Institute of Plant Physiology, Russian Academy of Sciences, Botanicheskaya Street 35, 127276 Moscow, Russia; 3 Department of Bioengineering, Faculty of Biology, Lomonosov Moscow State University, 1–12 Leninskie Gory, 119234 Moscow, Russia; 4 Institute of Natural Sciences, Derzhavin Tambov State University, Komsomolskaya Square 5, 392008 Tambov, Russia; 5 N.N. Semyonov Federal Research Center for Chemical Physics, Russian Academy of Science, 4 Kosygina Street, Building 1, 119192 Moscow, Russia

**Keywords:** phytohormone, microalgae, bacterial community, growth parameters, chlorophyll, carotenoids, fatty acid profile

## Abstract

Microalga *Chlorella (Chromochloris) zofingiensis* has been gaining increasing attention of investigators as a potential competitor to *Haematococcus pluvialis* for astaxanthin and other xanthophylls production. Phytohormones, including abscisic acid (ABA), at concentrations relevant to that in hydroponic wastewater, have proven themselves as strong inductors of microalgae biomass productivity and biosynthesis of valuable molecules. The main goal of this research was to evaluate the influence of phytohormone ABA on the physiology of *C. zofingiensis* in a non-aseptic batch experiment. Exogenous ABA stimulated *C. zofingiensis* cell division, biomass production, as well as chlorophyll, carotenoid, and lipid biosynthesis. The relationship between exogenous ABA concentration and the magnitude of the observed effects was non-linear, with the exception of cell growth and biomass production. Fatty acid accumulation and composition depended on the concentration of ABA tested. Exogenous ABA induced spectacular changes in the major components of the culture microbiome of *C. zofingiensis*. Thus, the abundance of the representatives of the genus *Rhodococcus* increased drastically with an increase in ABA concentration, whereas the abundance of the representatives of *Reyranella* and *Bradyrhizobium* genera declined. The possibilities of exogenous ABA applications for the enhancing of the biomass, carotenoid, and fatty acid productivity of the *C. zofingiensis* cultures are discussed.

## 1. Introduction

Among the most important goals of contemporary biology is the development of innovative technologies to meet the needs of society in food, pharmaceutical, and energy sectors. Green microalgae are promising objects for the production of alternative, environmentally friendly biomass rich in valuable molecules that can be used in various industries. Yet, the production of microalgae biomass is still not economically feasible in most cases. One of the promising ways to improve the feasibility of microalgal biomass-based industry is the use of efficient inductors of biomass productivity and biosynthesis.

The broad range of inductors used to enhance microalgae production includes, but is not limited to, the nutrient starvation method [1,2,3,4], salinity shifts [5,6], high light intensity [7,8], the use of heavy metals [9,10,11], as well as some organic substances application. Particularly, plant and animal hormones, which could be present in both surface and wastewaters, have been investigated for their impact on physiology of different microalgae species [12,13,14,15,16,17,18]. Notably, the research on the influence of hormones on microalgae physiology demonstrated simultaneous stimulation of biomass production and valuable biomolecule yields, which is very important for bringing biomass production to the industrial level [4,15,16,17,18].

The influence of phytohormones on microalgae physiology for biomass production is of particular interest for investigators for many reasons. First, microalgae and aquatic plants produce phytohormones [19,20,21] that efflux in trace concentrations into aqueous environments, where they affect algal cells [14,22,23]. Second, wastewaters from hydroponic cultivation in greenhouses and agricultural run-off from crops treated with plant growth regulators or hormone-herbicides have been considered as suitable, reach-in-nutrients media for algae biomass production [24,25,26]. Certainly, these types of wastewater can bear significantly higher than surface or groundwater concentrations of phytohormones, reaching hundreds of μM [27,28,29].

For instance, ABA, which has been detected in hydroponics wastewater in concentrations from hundreds of nM to µM [27,30], has proven itself as a strong inductor of microalgae physiology processes including growth and pigment biosynthesis and their accumulation in cells [14]. Primarily demonstrated in vascular plants, the main function of ABA, as a protective mediator against environmental stresses and pathogens, was confirmed in microalgae [23,31]. ABA is considered as an important two-way growth regulator, adjusting plant physiology to environmental stresses such as cold or hot weather, drought, the impact of pathogens, etc. [32,33,34]. In microalgae, ABA has been proven to increase growth parameters and pigment and lipid biosynthesis [13,14]. In vascular plants, ABA has been shown to participate in the regulation of carotenogenesis as a catabolite of neoxanthin and xanthoxin [35,36]. It is possible that ABA also plays an important role in microalgae carotenogenesis. Being an important agent for environmental stress mitigation, ABA has gained elevated interest in view of the application of hormonal induction of microalgae production in raceways or other open-type growth facilities. Moreover, phytohormones, working effectively in nanoconcentrations, have proven to be a promising and powerful tool for manipulating carotenogenesis and lipogenesis, which has been shown in some higher plants [34,36,37,38] and microalgae species [13,14,15,16].

A Chlorophyte *Chromochloris* (formerly *Chlorella*) *zofingiensis,* grown on an industrial scale for the production of carotenoids, is considered as a competitor to the major industrial producer of astaxanthin *Haematococcus pluvialis*. Compared to *H. pluvialis*, *C. zofingiensis* demonstrated two- to five-fold higher growth rate [39,40,41] and greater resistance to environmental stresses (primarily pathogens and pollutants), proving itself to be a robust species [42]. Although, the accumulation of astaxanthin in *C. zofingiensis* cells is, on average, six times less than in *H. pluvialis* cells [40,42,43,44,45].

Hence, to increase carotenoid production in *C. zofingiensis* cells so it will be comparable to that of *H. pluvialis* cells, a strong inductor of carotenogenesis must be applied. Importantly, this inductor should not compromise population growth parameters, while increasing biosynthesis and accumulation of carotenoids.

It is widely known that most of the microalgal strains exist in a close metabolic and regulatory relationship with bacterial species inhabiting their phycosphere [46]. The core microbiome of *H. pluvialis* cultures was studied in previous works and was shown to be affected under the conditions inducing carotenogenesis and lipogenesis [47,48]. It was discovered recently that the microbiome components could, in turn, affect the biomass production yield and astaxanthin biosynthesis rate in *H. pluvialis* under certain conditions [49]. Therefore, the possibility of the core microbiome modulation by phytohormones (e.g., ABA) is of great interest for the rational design of biotechnological processes in microalgae cultures, including *C. zofingiensis*.

In this study, to accomplish the goal of a simultaneous induction of growth and valuable molecule biosynthesis, ABA was chosen as an inductor with a range of concentrations relevant to that in surface water and hydroponic wastewater. At the same time, we monitored the possible effect of ABA concentrations on host–microbial interactions in *C. zofingiensis* culture. Accordingly, the experimental work with ABA was not aseptically conducted in order to test the growth of *C. zofingiensis* in the presence of an ambient microbiome to evaluate the use of hormonal induction in open raceway applications. Influence of ABA on *C. zofingiensis* was evaluated by comparing population growth, biomass production, chlorophyll-*a* and -*b* (Chl), total carotenoid (CR) and total fatty acid (TFA) production, optical absorbance of the pigment, and the fatty acid profiles with the control (0 μM ABA). Microbiome dynamics, in terms of taxonomic and quantitative analysis, in the non-aseptic experiment was assessed.

We found that exogenous ABA stimulated *C. zofingiensis* cell division, biomass production, as well as Chl, CR, lipid, and fatty biosynthesis in a non-linear concentration-dependent manner, with the exception of cell growth and biomass production. Exogenous ABA also induced spectacular changes in the major components of the culture microbiome of *C. zofingiensis*.

## 2. Materials and Methods

### 2.1. Strain and Culturing Conditions

The culture of *C. zofingiensis* (C-30) was obtained from the Microalgae and Cyanobacteria Culture Collection IPPAS (Timiryazev Institute of Plant Physiology, Russian Academy of Sciences; Moscow, Russia). *C. zofingiensis* was chosen as a potential alternative for *H. pluvialis* regarding production of keto-carotenoids, especially astaxanthin. The advantages of *C. zofingiensis* include higher culture robustness as well as high biomass, lipid, and CR productivities [40,50,51,52]. A stock culture of the algae was grown aseptically in standard Bold’s Basal Medium (BBM) [53] with NH_4_Cl added to the final concentration of 4.5 ppm (see the ions composition in Table S1) for one month prior to the experiment at 26 ± 1 °C, pH 6.9 ± 0.1, with white fluorescent lighting at 110 μmol/m^2^/s measured with a digital lux-meter TKA-Lux (NTP “TKA” Scientific Instruments, St. Petersburg, Russia) on an orbital shaker (JeioTech OS-7200, Daejeon, Republic of Korea). Prior to the experiment, the status of the culture was checked according to the health criteria identified by Environment Canada [54]: the standard curves of the Stock Culture growth and chlorophyll-*a* content were stable for two months each time the culture was restarted (every two weeks with a coefficient of deviation less than 10%); the culture was checked for bacterial and fungal contamination every month and before the experiment. This ensures the quality and relevance of the obtained data. The conditions of experimental work were the same as those used for the Stock Culture, with one exception: the ambient temperature during the experiment was 29 ± 1 °C. The seeding density of *C. zofingiensis* cells in all trials was 5.0 ± 1.0 × 10^4^ cell/mL.

### 2.2. Growth Media and the Treatments

#### 2.2.1. Range of the Hormone Concentrations and Working Solution Preparation

The range of hormone concentrations used in each experiment was chosen based on reported concentrations of ABA in surface water and in hydroponic wastewaters [26] as well as on the results of our previous experimental work [15]. ABA was obtained from Sigma-Aldrich Inc. (Missisauga, ON, Canada). The ranges of hormone concentrations tested were 1, 5, 10, and 50 μM, and ABA Stock Solutions were prepared in dimethyl sulfoxide (DMSO, Dia-M Ltd., Moscow, Russia). In our previous study on ABA influence on microalgae physiology, DMSO showed additional effect on algae cells when its concentration exceeded 0.5% of the Working Solutions (the final concentration of 500 μM and higher) [15]. Hence, in this study, we did not expect any influence of the solvent on the results. The Working Solutions for the test were prepared on the modified BBM. In the control trial, as well as in four trials with ABA concentrations, four biological replicates were used.

#### 2.2.2. Growth Media

The monitoring of general water chemistry and ambient conditions was performed daily: temperature and pH were measured using an HM DIGITAL pH-meter Com-300 (HM Digital Inc., Seoul, Republic of Korea). The pH of all the media tested was adjusted to 6.9 with 1 N HCl or 1 N NaOH solutions. All pH-adjusted media were equilibrated for 1 day prior to being used in the tests. The composition of dissolved compounds and ions in BBM is presented in Table S1 of the Supplementary Materials. Analyses of depletion of ions in BBM (as dissolved ions: Ca, Mg, K, Cu, Zn, and Fe) were conducted by atomic absorption spectrophotometry Shimadzu AA-7000 (Shimadzu, Kyoto, Japan) applying a hollow-cathode lamp (Hamamatsu Photonics, Shizuoka, Japan). Prior to the analyses, the culture medium samples were acidified with 0.1 M HNO_3_ (1% acidification). The pH of all the media tested was adjusted to 6.9 with 1 N HCl or 1 N NaOH solutions. All pH-adjusted media were equilibrated for 1 day prior to being used in the tests.

### 2.3. Sampling and Analytical Methods

#### 2.3.1. Algae Cell Counting

Cell density was determined by counting with a Goraev-chamber haemocytometer (Apexlab.ru, Moscow, Russia) using a Jenaval Carl Zeiss microscope (Carl Zeiss Jena, Jena, Germany) in 7 to 10 technical replicates. As the seeding density in each trial could not be absolutely equal, the proportional rate of increase between each time point was used to assess cell growth using the following formula:

Na = (Nt − N0)/t(1)

where Na is the calculated cell density, N0 is the initial cell density, and Nt is the cell density on day t.

#### 2.3.2. Assessment of Biomass Production

To determine the dry cell weight (dcw), a 20 mL aliquot of well-shaken culture was taken at the end of the experiment (day 11) from each flask into the centrifuge tubes. The cells were pelleted using a Sorvall™ RC 6 Plus centrifuge (Thermo Scientific™, Langenselbold, Germany) at 4500 rpm (2000× *g*) for 10 min. Then, the supernatant was discarded, and the cells were washed with 0.9% NaCl, pelleted again, dewatered, and oven-dried at 60 °C until constant weight.

#### 2.3.3. Assessment of Pigment Concentrations and Fatty Acid Profiling

Cells were pelleted by centrifugation, transferred to a glass-glass homogenizer with a chloroform-methanol (10 mL, 2:1, *v/v*) mixture and extracted to remove all pigment. The lipid fraction including Chl and carotenoids CR was separated according to Folch et al. [55]. The chloroform phase was used for further pigment and lipid analysis. Chl was quantified spectrophotometrically with the Agilent Cary 300 (Agilent, Santa Clara, CA, USA) spectrophotometer using absorption coefficients for chloroform [56]. Chlorophylls and total carotenoids were calculated using the method for methanol extraction as described by Eaton and Franson [42]. Chlorophyll, carotenoid, and FA concentrations per cell (ng/cell) were calculated using the following equation:

X = C/N × 1000(2)

where X = pigment concentration per cell in ng/cell, C = pigment concentration in µg/mL of extract, and N = number of cells per mL.

The spectra of the extracts were recorded using the same spectrophotometer. After completion of the pigment analysis, the chloroform was evaporated and the lipid residue was dissolved in methanol. Fatty acid (FA) methyl esters were prepared by transesterification of the lipids by refluxing for 1.5 h in methanol containing 5% conc. sulphuric acid [57] in the presence of 0.01% 2,6-di-tert-butyl-4-methylphenol as an antioxidant and heptadecanoic acid (C17:0) as an internal standard. Methyl esters were extracted with n-hexane and immediately used for GC analysis. The fatty acid methyl esters were separated and identified according to retention times of a 37-component FAME standard set (Supelco, Sigma, St. Louis, MO, USA) and by characteristic mass spectra obtained with an Agilent 7890 gas chromatograph equipped with a 30 m DB23 capillary column coupled with an Agilent 5970 mass-selective detector (Agilent, Santa Clara, CA, USA). Helium at a flow rate of 1 mL min^−1^ was used as a carrier gas. The FAs were classified on the basis of their CDW percentage either as major (>3% of total FA) or minor FA (<3% of total FA).

### 2.4. Analysis of the Microalgal Culture Microbiome

#### 2.4.1. 16 SrRNA Library Preparation for Metagenomic Analysis

For metagenomic analysis, from 50 mL of each fresh sample, a cell pellet was collected by centrifugation at 4500 rpm (2000× *g*) for 10 min and frozen at −80 °C immediately. Samples were stored for 3 months at −80 °C. For environmental DNA (eDNA) extraction, cell pellets were frozen in liquid nitrogen and grinded into a fine powder with homogenizing pestles (SSIBio Corp., Lodi, CA, USA) in a 1.5 mL microcentrifuge tube. The freezing-homogenization procedure was conducted repetitively three times for each sample. The eDNA was extracted from the obtained homogenates using a DNeasy Plant Pro kit (QIAGEN, Hilden, Germany) according to the manufacturer’s instructions. A purified eDNA was used as a template for polymerase chain reaction (PCR) with universal primers for amplification of the V4 region of the 16SrRNA gene [58], F515 (GTGCCAGCMGCCGCGGTAA) and R806 (GGACTACVSGGGTATCTAAT), from Bates et al. [59]. Sequencing was performed on a MiSeq platform (Illumina, Inc., San Diego, CA, USA) using a MiSeq Reagent Kit v3 (600-cycle) (MS-102-3003, Illumina, Inc., San Diego, CA, USA) for paired-end sequencing (2 × 300 bp).

#### 2.4.2. Metagenomic Data Analysis

Initial data processing, i.e., sample demultiplexing and adapter trimming, was carried out using the Illumina software v. 2.6 (Illumina, USA). Further procedures for denoising, combining sequences, restoring the original phylotypes (ASV, Amplicon sequence variant), deleting chimeric reads, and taxonomic classification of the obtained ASVs were performed in the R software v.4.2.2 environment using the dada2 [60], phyloseq [61], and DECIPHER [62] software packages, as well as the SSU 16s rRNA SILVA database (release 132) [63]. When analyzing the results of the amplicon sequencing, there were removed unclassified ASVs, singletone reads, and taxonomic groups with overall relative abundance lower than 0.1%.

α-diversity indices of Shannon and Simpson diversity and β-diversity indices of Morishita were calculated according to the formulas described in [64]. The boxplot with α-diversity indices and a heatmap with β-diversity indices of Morishita were visualized using the Python programming language (version 3.7.1) with the Matplotlib library. A Venn diagram was built using an online service (https://www.bioinformatics.psb.ugent.be, accessed on 15 December 2022). A clustered heatmap was plotted using an online service (https://www.bioinformatics.com.cn/en, accessed on 15 December 2022). 

### 2.5. Statistical Treatment

All trials were conducted using 4 to 5 independent biological replicates (*n* = 4 or 5) with 4–12 technical replicates, which were then analyzed for significant differences (*p* < 0.05) against the control (zero hormone concentration) using a one-way Analysis of Variance test (ANOVA). The significance of measured differences in biomass was assessed with a Mann–Whitney Rank Sum Test. Correlation analyses of the obtained data were performed using PCC (Pearson Correlation Coefficient). All the statistical analyses were performed using SigmaStat software (version 3.5, Systat Software, Inc., San Jose, CA, USA).

## 3. Results

### 3.1. Water Chemistry

The results of monitoring the changes in the composition of dissolved ions demonstrated that ABA concentrations influenced depletion of the cations in the growth medium (Table 1). Among the tested ABA concentrations, the order of influence was 50 > 1 > 5 ≥ 10 µM for Ca, Mg, and Zn, and 50 > 10 ≥ 5 ≥ 1 µM for K and Fe. The ion composition also depended on the duration of the experiment. The ion concentrations followed different patterns: in the control, the tested ions did not show a significant change, except for Mg and Fe ion depletion on the 10th and 16th days of the experiment. The key cations Ca and Mg were significantly depleted by the end of the experiment at all the tested ABA concentrations except 10 µM for Ca (Table 1). Only the highest tested ABA concentration of 50 µM was capable of depleting K by day 10 of the experiment and further. Importantly, significant increases in concentration were observed for Fe (on day 7 at 5 and 10 µM; on day 10 at 1 and 5 µM), for K (on day 7 at 5 µM and on day 10 at 1 µM), and for Zn on day 10 (5 and 10 µM).

By the end of the experiment (day 16), the pH of the growth media in all the trials shifted to the alkaline conditions of 8.3 ± 0.5, as is normally observed during the green algae population growth. No significant difference in the pH change was observed in comparison to the control or among the tested ABA concentrations.

### 3.2. Effect of ABA on C. zofingiensis Growth and Biomass Production

**Figure 1:**
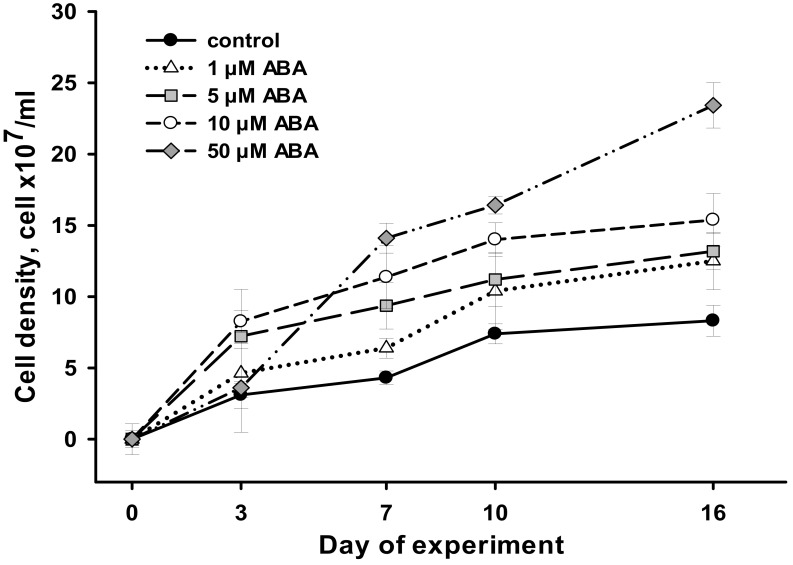
Cell density of *Chlorella zofingiensis* cultured in a range of ABA concentrations of 1 µM to 50 µM during 16 days of experiment. Data are shown as the mean ± SD, *n* = 12. Control cultures did not contain ABA.

The induction effect of ABA on *C. zofingiensis* cell division was evident at all the tested concentrations, starting from the seventh day of exposure, and further compared with the control (Figure 1). Earlier, only 5 μM and 10 μM ABA exerted sizeable effects.

On day 16 (the end of the experiment), the greatest promotion of *C. zofingiensis* cell density (2.8-fold greater than in the control) was observed at 50 μM ABA (Table 2). The ABA concentration of 10 μM gave a 1.9-fold increase in density, while 1 and 5 μM ABA demonstrated similar effects (a 1.6-fold increase in cell density; Figure 1). The effects of the ABA concentrations ranging from 1 to 10 μM on cell density of the algae became similar at the end of the experiment (Table 2).

While all the tested ABA concentrations were capable of increasing biomass production of *C. zofingiensis*, the greatest effect was obtained at 50 μM ABA (a 3.4-fold increase dcw at the end of the experiment; Table 2). Overall, the magnitude of the effect of ABA on cell density and biomass production was directly proportional to the ABA concentration.

### 3.3. Effect of ABA on Pigment Production in C. zofingiensis

#### 3.3.1. Chlorophyll Content in the Cells

Chl content of *C. zofingiensis* cells was significantly elevated by all tested ABA concentrations, but the effect was concentration-dependent (Table 2). On the 3rd day of the experiment, a significant increase in Chl/cell was observed in the presence of 1 and 50 μM ABA. Remarkably, the stimulatory effect remained on day 7 only in the 1 μM ABA, while 50 μM had declined Chl content as compared to the control on day 7 and further. By the end of the experiment, 5 and 10 μM ABA increased the concentration of Chl per cell, whereas 50 μM ABA retained the inhibitory effect on Chl. The most profound effect on Chl was observed at 50 μM ABA (day 3, 2.3-fold increase relative to control) and 1 μM ABA (day 7, 2.2-fold), translating into 418 fg/cell and 410 fg/cell of Chl, respectively. Regarding the stimulatory effect on Chl, the ABA treatments were ordered as follows: 1 > 50 > 5 > 10 μM (Table 2). In the control (no ABA added), the concentration of Chl/cell was only slightly elevated at the end of the experiments (Table 2). If considering production of chlorophyll, all the tested ABA concentrations significantly induced the parameter at the end of the experiment. The highest influence on Chl production was demonstrated in 1 μM ABA with an increase of 2.7-fold. The order of efficacy of ABA for Chl production was 1 > 5 > 50 ≥ 10 μM.

#### 3.3.2. Cell Content of Total Carotenoids

The total CR content per cell was increased profoundly on the third day of the experiment by the lowest and the highest concentration of ABA (1 and 50 μM, 1.6- and 1.9-fold increase or 133 and 159 fg/cell, respectively). Similarly to Chl accumulation, the greatest effect was observed on the third and seventh day of the experiment, with a subsequent mitigation of the effect (Table 2). The concentration of 10 μM ABA was adverse to the CR content of the cells, while 5 μM ABA exerted no effect. The order of the efficacy for CR cell content stimulation was 50 > 1 > 5 > 10 μM ABA. No significant increase in CR per cell was observed in the control (Table 2). The influence of ABA on CR production was in the following order: 50 ≥ 1 > 5 μM, while 10 μM ABA did not affect the production (Table 2). The greatest 2-fold increase in CR production compared to the control was detected at 50 μM ABA.

In the control trial, both ratios, TFA/Chl and TFA/CR, were stable in time. The highest values of TFA/Chl and TFA/CR were observed at 50 μM, and slightly lesser at 1 and 5 μM ABA on day three of the experiment. For the TFA/Chl ratio, the maximum values were on the 7th and 16th days (50 μM ABA) due to the lowest Chl concentrations per cell on these days.

#### 3.3.3. Optical Absorbance Spectra of the Cell Pigment Extracts

**Figure 2:**
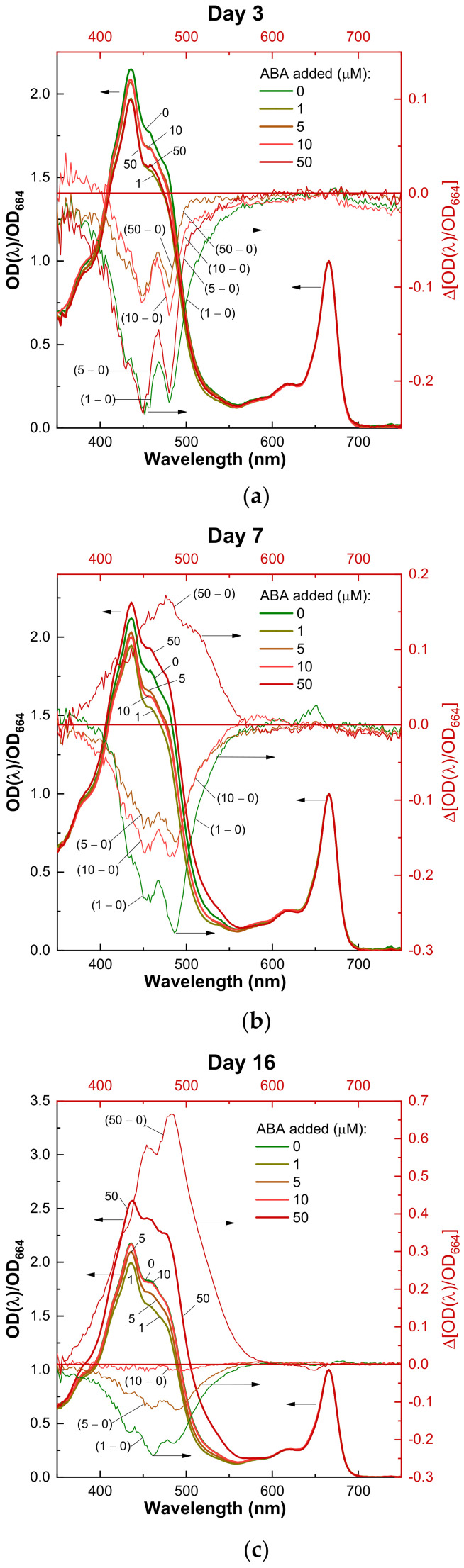
Representative changes in optical absorbance spectra of the pigment extracts (see Methods) from the cells of *Chlorella zofingiensis* incubated for (**a**) 3, (**b**) 7, or (**c**) 16 days in the presence of different ABA concentrations. The spectra normalized to the red Chl absorption maximum (left scale) are shown together with the difference spectra (right scale) obtained by subtracting the control (0 µM ABA) spectrum from the spectrum of a corresponding ABA treatment. The ABA concentrations are shown on the graphs.

In addition to the analysis of the total CR content, we studied the changes of the normalized absorbance spectra of the pigment in DMSO extract of the *C. zofingiensis* cells exposed to different ABA concentrations in order to reveal the trends of the CR composition change. In line with the dynamics of the CR culture content depicted above, the cultures after 3-day incubation in the presence of ABA displayed a decline in the relative amplitude of the absorption bands in the blue-green region of the visible part of the spectrum. Overall, the cultures exposed to ABA concentrations showed a sizeable decline in the absorption bands attributable to the primary (photosynthetic) CR, apparent as negative peaks on the difference absorbance spectra (Figure 2A, right scale). This trend was also documented in the cultures exposed to ABA for 7 days with a single exception. Namely, the culture exposed to 50 µM ABA demonstrated an increase in the relative absorption of the pigments with maxima located in the band of 480–490 nm (Figure 2B, right scale), resembling the absorbance spectra of secondary (keto-)carotenoids commonly found in microalgae [65]. These changes became the most remarkable after 16 days of the experiment (Figure 2C).

### 3.4. The Effect of ABA on Fatty Acid Composition of C. zofingiensis Cells

All the tested ABA concentrations stimulated FA accumulation in *C. zofingiensis* cells. TFA concentration per cell depended on both the phytohormone concentration and the duration of exposure (Table 2). A significant increase (1.2-fold at maximum) of TFA was also observed in the control. Remarkably, in all the tested ABA treatments, the maximum induction of TFA was detected on day three of the experiment, with subsequent significant mitigation of the effect at the end of the experiment (Table 2). The greatest increase in TFA concentration was noted in the 1 and 50 μM ABA treatments (3.0- and 4.8-fold, respectively). The order of ABA stimulation efficiency on TFA synthesis on day 3 was 50 > 1 > 5 μM, while 10 μM displayed no significant induction and further declined the TFA content. On days 7 and 16, the order was 1 > 50 μM, while 5 and 10 μM ABA demonstrated an adverse effect on TFA synthesis.

Production of TFA was induced by all the tested ABA concentrations (Table 2). The most profound effect on TFA accumulation was detected at 50 μM ABA, resulting in a 5.6-fold increase. The order of ABA stimulation efficiency on TFA production at the end of exposure was 50 > 1 > 10 ≥ 5 μM (Table 2).

**Figure 3:**
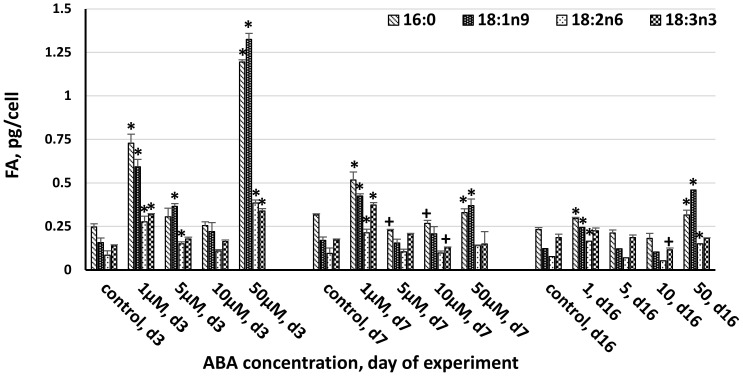
The effect of abscisic acid on the composition of major fatty acids (>3% of total FAs) in the cell of *Chlorella zofingiensis* on the 3rd, 7th, and 16th days of experiment (d3, d7, and d16, respectively). Statistically significant differences from the controls (*p* < 0.05) are indicated, where *—significantly greater than the control and +—significantly lower than the control. Data are shown as the mean ± SD, *n* = 4. Control cultures did not contain ABA.

**Figure 4:**
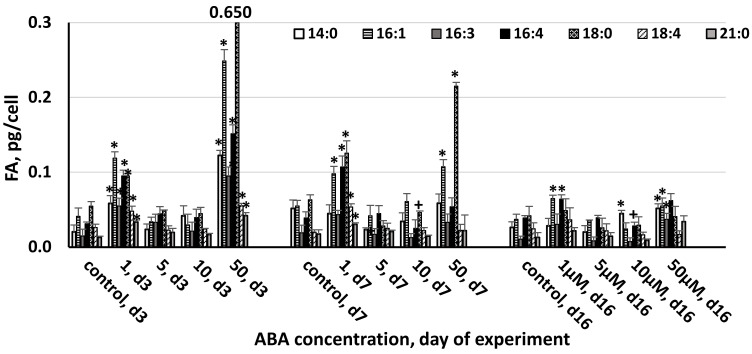
The effect of abscisic acid on the composition of minor fatty acids (1–3% of total FAs) in the cell of *Chlorella zofingiensis* on the 3rd, 7th, and 16th days of experiment (d3, d7, and d16, respectively). Statistically significant differences from the controls (*p* < 0.05) are indicated, where *—significantly greater than the control and +—significantly lower than the control. Data are shown as the mean ± SD, *n* = 4. Control cultures did not contain ABA.

The FA profiles were strongly influenced by ABA concentration and the duration of exposure (Figure 3 and Figure 4). The greatest increase in the accumulation of major fatty acids (>3% of TFAs) was observed at 50 μM ABA, with palmitic (C16:0), oleic acids (C18:1n9c), linoleic acid (C18:2n6), and α-linolenic acid (C18:3n3) demonstrating their maximum concentrations of 1.195, 1.324, 0.385, and 0.339 pg/cell, respectively (Figure 3). Among the tested ABA concentrations, on the 3rd and 16th day of the experiment, the order of efficacy was 50 > 1 > 5 μM, while 10 μM was ineffective or even adverse to certain FAs. On day seven, 1 μM ABA was more effective compared to 50 μM ABA, while 5 and 10 μM ABA demonstrated no effect or a negative effect on FA. The same concentration of ABA had a different influence on a single major FA. Thus, palmitic and oleic acids were the most responsive to the induction by ABA (4.9- and 8.4-fold increase at 50 μM and 2.9- and 3.8-fold increase at 1 μM on the third day of the experiment).

Minor FAs were also most affected by 1 and 50 μM ABA. Similarly to the major FAs, the effect of ABA on their synthesis was mitigated toward the end of the experiment. The greatest increase in the accumulation of the minor fatty acids (1–3% of TFA) was observed at 50 μM ABA on the third day, with myristic (C14:0), palmitoleic (C16:1), cis-hexadecatrienoic (C16:3), hexadecatetraenoic (C16:4), octadecanoic (C18:0), stearidonic (C18:4), and heneicosanoic (C21:0) acids demonstrating their maximum concentrations of 0.123, 0.249, 0.095, 0.650, 0.055, and 0.0421 pg/cell, respectively (Figure 4).

**Figure 5:**
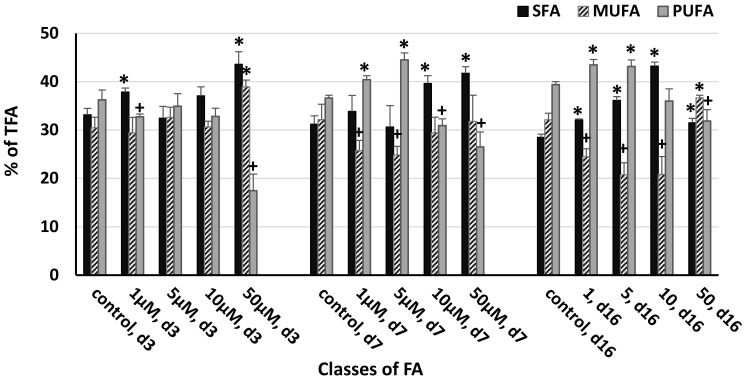
The influence of abscisic acid on the distribution of fatty acid classes in the cell of *Chlorella zofingiensis* during the experiment on the 3rd, 7th, and 16th days of the experiment (d3, d7, and d16, respectively). Statistically significant differences from the controls (*p* < 0.05) are indicated, where *—significantly greater than the control and +—significantly lower than the control. Data are shown as the mean ± SD, *n* = 4. Control cultures did not contain ABA.

Considering the classification of FAs, it should be noted the following: in control samples, the distribution of classes was similar on days 3 and 7 (PUFA > SFA ≥ MUFA), and, toward the end of experiment, the dominance of PUFA over other classes was evident (Figure 5). When *C. zofingiensis* cells were exposed to the range of ABA concentrations, the differences in the distribution of classes were apparent. The percentage of SFA was elevated at 1 and 50 μM ABA on day 3, at 10 and 50 μM ABA on day 7, and at all the tested ABA concentrations at the end of the experiment (day 16). The percentage of MUFA was increased only at 50 μM ABA (on days 3 and 16), while 1 and 5 μM ABA (on day 7) and 1, 5, and 10 μM ABA (on day 16) significantly lowered its percent. PUFA fraction increased at 1 and 5 μM ABA (on days 7 and 16) and declined at 1 μM (on day 3), 10 μM (on day 7), and 50 μM (at any sampling day). In other cases, no effect of ABA on the classification of FAs was observed compared to the control.

### 3.5. Effect of the ABA Treatment on the Microbiome of C. zofingiensis Culture

**Figure 6:**
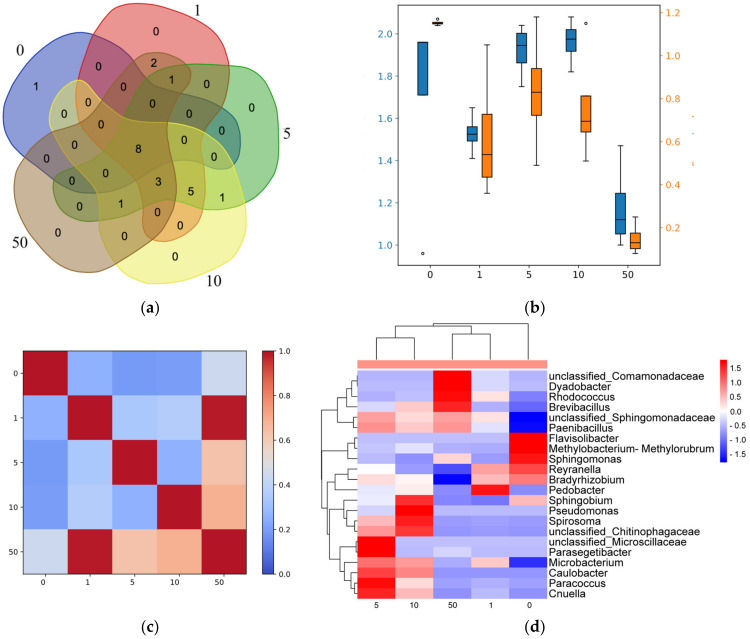
Metagenomic analysis of the *Chlorella zofingiensis* cultures incubated for 16 days in the presence of different ABA concentrations: 0—0 μM, 1—1 μM, 5—5 μM, 10—10 μM, 50—50 μM. Interception between microbiomes of cultures is presented as a Venn diagram (**a**). The box plot represents mean values (*n* = 4) of microbial community biodiversity (Shannon index, blue) and evenness (Simpson index, orange) in studied cultures (**b**). Comparison of the cultures’ similarity is estimated by the Morisita index of β-diversity and depicted as a heatmap (**c**). Cultures’ microbiomes similarity was observed on a genus level on a clustered heatmap (**d**).

The prokaryotic composition in the *C. zofingiensis* culture was investigated by the metagenomic sequencing of the 16S rRNA amplicons on day 16. At the end of exposure, the cultures shared a highly similar list of taxa among different concentrations of phytohormone ABA (Figure 6A). Among intercepting genera of bacteria were found *Rhodococcus*, *Reyranella*, *Methylobacterium-Methylorubrum*, *Bradyrhizobium, Flavisolibacter, Sphingomonas, Sphingobium, Microbacterium,* and *Paenibacillus*. Despite that, differences were observed in relative abundance of the genera for various concentrations of ABA, which resulted in α-diversity indices (Figure 6B). According to the Shannon index, none of the samples demonstrated higher values of biodiversity than non-treated culture. The concentrations of 5 and 10 μM ABA did not affect the biodiversity at a significant level. In contrast, concentrations of 1 and 50 μM ABA led to a 1.3- and 1.7-fold decrease of biodiversity, respectively. At the same time, the genera distribution evenness in the microbiomes was dramatically shifted by ABA treatment (Figure 6B). The Simpson diversity index was shown to be decreased 2-fold for 1, 5, and 10 μM ABA concentrations, and 8-fold for 50 μM ABA, which indicates the overdomination of the microbial population by a single species. The abundance of genus *Rhodococcus* was positively affected by increasing the concentration of ABA and consisted approximately 70% of the whole diversity for 50 μM ABA, while *Reyranella* and *Bradyrhizobium* were decreased almost 2-fold (Table 3).

The comparison of the cultures by the Morisita index of β-diversity confirmed observations of α-diversity trends (Figure 6C). The cultures treated with different concentrations of ABA showed high similarity with the highest concentration 50 μM ABA. Remarkably, the similarity between the pair of microbiomes treated with 1 and 50 μM ABA was higher than between others. To thoroughly investigate that finding, further comparison of the cultures’ microbiomes was conducted by clustering with the visualization on the heatmap (Figure 6D). As was already expected, the cluster analysis determined three subgroups of samples: non-treated culture stands separate, cultures treated with 1 and 50 μM ABA, and cultures treated with 5 and 10 μM ABA. The heatmap shows that genera *Reyranella* and *Bradyrhizobium* form a common cluster that is represented in control culture, as well as *Methylobacterium* and *Sphingomonas*. Another cluster is formed within group of samples treated with 1 and 50 μM ABA, which consisted of *Rhodococcus*, *Paenibacillus*, *Dyadobacter,* and *Brevibacillus*. Cultures treated with 5 and 10 μM ABA formed a cluster of microbiome genera *Pseudomonas*, *Spirosoma*, *Microbacterium*, *Caulobacter*, *Paracoccus,* and *Cnuella*.

## 4. Discussion

Application of phytohormones in low concentrations for simultaneous and significant induction of biomass and biomolecules production opens up great opportunities for reducing the cost of microalgae biomass production, making it economically viable. Moreover, the use of hydroponics wastewater as a source of phytohormones contributes to the solution of two important tasks in view of industrial integration: effective biomass production and wastewater treatment. However, phytohormone applications as the inductors of microalgae productivity are poorly studied, and previous results have to be proven for a wide range of species of industrial interest. To date, only a few studies, including our research on *Scenedesmus quadricauda*, have been conducted on microalgae using ABA in low concentrations relevant to that in hydroponic wastewater [13,14,15,66].

ABA was chosen for experimental work in this study for two main reasons. First, this hormone plays an important role in plant cell immunity, inducing or reducing defense responses depending on the type of stress or pathogen [67,68], which is important when growing algae in open facilities. Second, ABA is the hormone that has been shown to be a supreme inducer of biosynthesis and accumulation of lipids and CR in microalgae [13,15,69,70].

Selection of a species for experimental work is an important question and must be clearly motivated. *C. zofingiensis* can be considered as the most promising producer of astaxanthin and other xanthophylls. This microalga can compete with *H. pluvialis* for dominance on an industrial scale of production of astaxanthin. However, a strong method of induction of the target compounds’ biosynthesis in *C. zofingiensis* cells must be developed. Most used methods of induction on an industrial scale are nitrogen limitation [40,71] and acetate application [42], or their combinations [43]. These methods can give quite high numbers of photoautotrophic biomass production, up to 7–10 g/L [40,43], which is about three times greater compared to our result on ABA induction. However, it must be kept in mind that, in contrast to our results, the maximum biomass production and the maximum pigment yield are usually achieved at different inductor concentrations when common industrial methods are applied [40,43,71], confirming the conflicting relationship between elevated biomass production and induction of biosynthesis of secondary metabolites. Moreover, it should be noted that our experiment was conducted not aseptically, which makes it possible for the microbiome to absorb and biodegrade some portion of aqueous ABA, which will be discussed later. It is equally important to emphasize that the ambient temperature was elevated (27–32 °C) during our experiment. On this issue, Del Campo et al. [40] observed dramatic suppression of CR accumulation (1.7-fold as pg/cell) with an increase of ambient temperature to above 28 °C.

The most effective ABA concentrations in our experiment (1 and 50 mM) greatly increased all the tested parameters including biomass, TFA, Chl, and CR production by the end of the experiment. Although, it has to be noted that the maximum accumulation of pigments and TFA under ABA influence occurred shortly after the beginning of exposure (on day 3 of the experiment), when the cell density was not sufficient for biomass harvesting. The mitigation of the effect up to the adverse influence of ABA at the end of the test could be explained by the degradation of ABA in the batch experiment. Likely, the products of ABA degradation do not contribute to induction of *C. zofingiensis* biosynthesis and/or could be toxic to the algae. A similar kinetics of mitigation of induction was observed in previous studies on phytohormones [15,16,72,73] and different nitrogen sources [65]. Thus, the results of a batch experiment on a phytohormone influence on microalgae physiology should be confirmed in a feed-batch or continuous experiment.

Our results, obtained on *C. zofingiensis*, are consistent with the previous reports on different microalgae species revealing the ability of ABA to induce growth parameters, biomass production and population growth, with a high correlation coefficient (Table S2). Yet, some discrepancies have been observed in the reports, as well as between the present study and our previous investigation. A recent study [74] demonstrated statistically significant but weak induction of *Chlorella* sp. population growth (1.2-fold increase at maximum) when 38 µM ABA was applied to the growth medium. However, the authors discussed the species-specific and possibly strain-specific response of microalgae cells to hormones. Wu et al. [70] also found the strain-specific influence of ABA on *Chlorella* when the biomass production of FACHB31 strain was not affected by ABA in the range of 0.4 to 75 µM, while the biomass yield of ZF strain was promoted and increased by 1.4-fold at maximum. Fiery et al. [66] examined low ABA concentrations of 0.5 to 5 µM, which are close to the most effective ABA concentration of ABA in our study, and observed a weak induction of ABA on biomass yield of *Phaeodactylum tricornutum* and no effect on *Stauroneis* sp and *Nitzschia* sp. Contrary to some works on different *Chlorella* species [15,70,74], no effect on growth parameters of *C. vulgaris* was observed [75] when comparable to our range ABA concentrations were applied. Most likely, this outstanding result occurred because ABA was applied on day 20 of the population growth (early stationary phase), highlighting the importance of the hormone application in the exponential phase of growth or even in the lag phase.

Such strong discrepancies between the impact of ABA on different species originate from the differences in the chemical composition of a growth medium and, certainly, due to the structural differences in cell walls, as discussed for biomass yield. Our results on the dynamic of cations in the growth medium during the experiment strongly support these assumptions. The dynamic of the major ions (Ca, Mg, and Zn) during the experiment revealed that the most effective concentrations of ABA for the induction of TFA, CR, and Chl also strongly affected the exchange of these cations through the membrane of the *C. zofingiensis* cells. The exogenous ABA application can promptly increase cytosolic Ca^2+^ via membrane Ca^2+^-channels [76]. It can be assumed that cytosolic Ca^2+^, being a typical intracellular second messenger for a number of signaling pathways, regulates the cell membranes’ permeability through ion channels. Therefore, elevated cytosolic Ca^2+^ concentration can increase the inward-directed ion current controlled by the ABA signal [77,78,79], which results in increasing some ions in the surrounded medium. However, our results demonstrated that this inward-directed efflux is selective and depends on the type of ion and ABA concentration.

Comparing the current results obtained on *C. zofingiensis* with our previous study on ABA influence on *S. quadricauda* physiology [15], the growth parameters of *C. zofingiensis* were much more strongly promoted by equivalent concentrations of ABA when both species were grown in identical medium (1.6- and 2.8-fold, respectively, as maximum cell density in BBM). This difference may be associated with the elevated T °C, the presence of a microbial community, and/or with differences in the structure of cell membranes and, therefore, their permeability to ABA of the algae species in question [71,80,81], as was discussed above.

Our research revealed significant induction of pigment and lipid biosynthesis and accumulation of these compounds in the *C. zofingiensis* cells, and these observations are in line with the processes reported in microalgae cells [82,83]. However, the degree and the kinetics of the induction of these biomolecules’ accumulation depended on ABA concentration and were somewhat different from the induction of the growth parameters. Unlike the population growth and biomass yield of *C. zofingiensis*, accumulation of the tested biomolecules demonstrated nonlinear dependence on the ABA concentrations. This pattern was also observed in relation to the effect of other phytohormones on the production of lipids and pigments [15,66,84] and a well-known effect in pharmaceutics [85,86]. Clearly, this pattern is also species-specific [66,87]. Thus, a wider range of phytohormone concentrations needs to be studied to clarify the optimal concentration for the induction of the target metabolites in each algae species of industrial interest. Therefore, our results indicated that the most effective concentrations of ABA for the induction of Chl, CR, and TFA production, as well as their concentrations per cell, are 1 and 50 µM. In support of that, the effect of the highest concentration of ABA (50 µM) used in this work was expressed in a pronounced increase in the pigments whose absorption bands are close to the absorption bands of known secondary CR [88]. Although, 5 and 10 µM also demonstrated effectiveness shortly after the exposure to the hormone (TFA) or to the end of the exposure (Chl). At the same time, it is important to remember that the population growth was significantly stimulated by all the tested concentrations of ABA after the 7th day of the experiment. The adverse effect of ABA on Chl, CR, and TFA concentration per cell was noted toward the end of the exposure in concentrations of 5 to 50 µM. However, only 10 µM ABA significantly reduced CR biosynthesis, so its production was close to the control. Thus, great induction of growth parameters by ABA was sufficient to overcome the adverse effect of the hormone on biosynthesis of the tested biomolecules, resulting in the elevated values of the molecules’ production (e.g., TFA production at 5 and 10 µM ABA). However, further research on the influence of the products of ABA degradation and the behavior of ABA in a feed-batch experiment would prove to be an asset for better understanding of the hormone influence on microalgae physiology.

Other investigators have also reported the great ability of ABA to induce lipids and pigments. Park et al. [13], when comparing the influence of five phytohormones on the physiology of *Chlamydomonas reinhartdii,* observed a 1.6-fold increase in the concentration of TFA at 38 µM ABA and a 1.9-fold increase in Chl, while no effect on biomass was detected. It is noteworthy that the effect of ABA on FA accumulation in their study was the greatest among the tested hormones. This is perfectly consistent with our findings on *C. zofingiensis* and on *S. quadricauda* [15], where ABA demonstrated a remarkable effect on FA accumulation with the highest values of palmitic (C16:0) and oleic acids (C18:1n9c). Although, in contrast to the results of Kozlova et al. [15], the presented study, as well as the results of Park et al. [13], demonstrated a powerful influence of ABA on pigment (Chl and CR) biosynthesis. The effect of ABA on distribution of different FA classes in *C. zofingiensis* cells appeared to have some discrepancies with the previously reported data. Lin et al. [74] observed a substantial increase in the percentage of MUFA, no effect on SFA, and a clear decline in the percentage of PUFA on the 10th day of their experiment when *Chlorella* sp. was exposed to an ABA range similar to ours. In another work on *C. vulgaris* treated with a close range of ABA concentrations, the percentage of SFA greatly increased and the fraction of PUFA decreased, but no effect was detected on the MUFA fraction [75], although, in this study, sampling was performed on the second day of exposure to ABA (the 22nd day of the experiment). Different induction strategies also resulted in a decline of the PUFA fraction and a great MUFA induction at the end of the experiment (15th day) when high light and nitrogen limitation were used [89]. In our study, all the tested ABA concentrations increased the SFA percentage, only 50 µM ABA increased the MUFA fraction, and, surprisingly, 1 and 5 µM ABA increased the PUFA fraction on the day of the test termination. Such significant differences can be the result of a number of reasons. We have already mentioned some of them, such as species differences and/or the chemical composition of the growth medium. It is quite possible that the duration of exposure to the inductor modulates the distribution of FA classes in microalgae cells. Indeed, Minyuk et al. [52] observed a clear difference in the distribution of TFA fractions between green (17 days of growth) and red cells (15 days of induction) of *C. zofingiensis*, while the influence of the N-source on the distribution was not evident. It is important to note that, on the 17th day of their experiment (green stage), the distribution of TFA fractions in the control was similar to our result at the end of the experiment (day 16). Our results evidently demonstrate changes in the allocation of TFA classes depending on the duration of exposure. These features found by us are important for the industrial production of algal FAs.

The link between lipid and carotenoid production has been defined in several studies [83,90,91,92,93]. Accumulation of CR strongly depends on biosynthesis and accumulation of cell lipids and, hence, TFA, but not vice versa [93,94]. The stable in time ratios of TFA/Chl and TFA/CR suggest balanced production of fatty acids and the pigments, regardless of the likelihood of nutrient depletion during the 16 days of the batch experiment when no ABA was applied. Unlike the influence of light intensity and nitrogen starvation [83], ABA in the range of 1 to 10 µM was capable of inducing both Chl and CR to a greater extent than TFA, which resulted in a decrease in both the TFA/Chl and TFA/CR ratios. This is confirmed by the PCC analysis (Table S2).

Another great concern about the application of the stress-related phytohormone ABA for the induction of pigment and lipid synthesis lies within its possible impact on the host-associated bacteria, which are known to play a crucial part in microalgal culture stability and productivity [95]. For the agricultural plants, it was shown that stress conditions highly affect microbial composition of the rhizosphere [96,97]. On the other hand, phytohormones are able to not only positively affect plant physiological state, but also stimulate certain bacterial genera in the microbiome of the roots of higher plants, as a putative carbon source. Previously, it was reported that plant growth-promoting bacteria (PGPB) are capable of degradation and utilization of certain phytohormones: ethylene, salicylic acid, indole-3-acetic acid, etc. [98,99]. ABA is known to be catabolized by *Rhodococcus* sp. P1Y down to dehydrovomifoliol [99]. In the current study, the relative abundance of genus *Rhodococcus* grew in response to the ABA increase. Simultaneously, the increase of ABA concentration in cultures suppressed the relative abundance of genus *Bradyrhizobium*, which is known as a natural producer of various phytohormones in the rhizosphere and is capable of inducing an immunity response in plants to protect them from phytopathogens [100,101]. The authors of the current study also noticed the microbiomes from cultures treated with the minimal and maximal concentrations of ABA (1 and 50 μM, respectively) had high relative abundances of PGPB genera, such as *Paenibacillus*, *Dyadobacter,* and *Brevibacillus*, which might increase macronutrient availability and are important elements of host–microbial interactions in plants–rhizosphere and microalgae–phycosphere systems [102,103,104]. On the other hand, the middle range concentrations of ABA (5 and 10 μM) resulted in stimulating the growth of the genera, some of which are known to accompany microalgal cultures in the photobioreactor systems and provide microalgae with organic substrates or act as parasitic organisms [48,105].

## 5. Conclusions

Exogenous ABA exerted a pronounced effect on all tested parameters of the chlorophyte *C. zofingiensis*. The magnitude of these effects depended on the ABA concentration, and, for certain parameters, was not stable over time: the effects on TFA, CR, and Chl were mitigated by the end of the experiment. Some of the tested ABA concentrations possessed an adverse effect on the microalga. Overall, these results demonstrate the necessity of further clarification of ABA effect either in the feed-batch or in the continuous cultivation mode. The capability of ABA to induce the accumulation of valuable biomolecules without compromising the growth of *C. zofingiensis* is important for its industrial production. The characteristic of the microbiome of ABA-treated *C. zofingiensis* cultures is also important for the industrial application of phytohormones as inductors of biomass and valuable molecule production. We conclude that ABA is a promising inductor for the industrial production of *C. zofingiensis* biomass, pigments, and lipids. Yet, more research is needed for clarification of the optimal hormone concentrations, time of harvesting, and the relationship between optimal ABA concentrations and ambient conditions of the cultivation unit.

## Figures and Tables

**Table 1:** Ion composition (ppm) in the growth medium during 16 days of the experiment.

Ion, ppm	Day of Experiment	ABA, µM				
		0	1	5	10	50
Ca	3	9.28 ± 0.26 ^aA^	8.10 ± 0.31 ^aB^	7.98 ± 0.44 ^aB^	7.56 ± 0.46 ^aB^	7.17 ± 0.15 ^aB^*
	7	8.53 ± 0.37 ^aA^	6.78 ± 0.59 ^bB^	8.65 ± 0.24 ^aA^	7.96 ± 0.49 ^aA^	6.42 ± 0.27 ^bB^
	10	8.51 ± 0.68 ^aA^	5.14 ± 0.94 ^bB^	8.26 ± 1.27 ^aC^	7.57 ± 0.68 ^aD^	4.76 ± 0.30 ^cF^
	16	8.42 ± 0.85 ^aA^	5.15 ± 1.06 ^bB^	6.09 ± 0.66 ^bB^	6.39 ± 0.81 ^aB^	4.96 ± 0.51 ^cB^
Mg	3	8.21 ± 0.32 ^aA^	7.24 ± 0.28 ^aB^	8.59 ± 0.16 ^aB^	7.56 ± 0.23 ^aB^	6.95 ± 0.07 ^aB^
	7	7.78 ± 0.18 ^aA^	6.90 ± 0.31 ^bB^	8.35 ± 0.57 ^bB^	7.08 ± 0.31 ^bB^	7.08 ± 0.16 ^aB^
	10	7.32 ± 0.30 ^bA^	6.57 ± 2.65 ^bB^	6.78 ± 0.76 ^bB^	6.43 ± 0.38 ^bB^	4.79 ± 0.17 ^aB^
	16	7.50 ± 0.24 ^bA^	6.18 ± 0.42 ^cB^	6.85 ± 0.25 ^cC^	6.70 ± 0.19 ^bA^	4.56 ± 0.22 ^bD^
K	3	107.17 ± 3.61 ^aA^	89.37 ± 4.68 ^aB^	86.09 ± 2.83 ^aB^	83.02 ± 2.64 ^aB^	73.06 ± 2.66 ^aC^
	7	109.87 ± 10.85 ^aA^	87.08 ± 1.06 ^aB^	94.03 ± 3.79 ^bAB^	86.72 ± 7.88 ^aA^	75.61 ± 4.40 ^aBC^
	10	107.47 ± 4.95 ^aA^	101.66 ± 5.64 ^a^*^A^	92.60 ± 5.66 ^aA^	64.72 ± 5.44 ^bB^	53.54 ± 3.00 ^bC^
	16	110.05 ± 5.45 ^aA^	80.71 ± 8.59 ^aB^	79.83 ± 6.82 ^a^*^B^	77.85 ± 6.78 ^abB^	58.26 ± 2.74 ^bC^
Fe	3	0.78 ± 0.008 ^aA^	0.59 ± 0.005 ^aB^	0.47 ± 0.04 ^aB^	0.39 ± 0.044 ^aA^	0.17 ± 0.021 ^aB^
	7	0.80 ± 0.063 ^aA^	0.64 ± 0.11 ^aA^	0.58 ± 0.14 ^bA^	0.60 ± 0.031 ^bA^	0.39 ± 0.053 ^bB^
	10	0.70 ± 0.042 ^bA^	0.67 ± 0.041 ^bA^	0.57 ± 0.09 ^bA^	0.48 ± 0.013 ^bB^	0.20 ± 0.048 ^aC^
	16	0.68 ± 0.012 ^bA^	0.56 ± 0.052 ^aA^	0.50 ± 0.023 ^cB^	0.41 ± 0.00 ^aC^	0.23 ± 0.01 ^bD^
Zn	3	0.19 ± 0.021 ^aA^	0.14 ± 0.034 ^aA^	0.16 ± 0.035 ^aA^	0.16 ± 0.013 ^aA^	0.112 ± 0.012 ^aB^
	7	0.15 ± 0.028 ^aA^	0.13 ± 0.021 ^aA^	0.16 ± 0.038 ^aA^	0.18 ± 0.023 ^aA^	0.15 ± 0.043 ^aA^
	10	0.18 ± 0.025 ^aA^	0.16 ± 0.03 ^aA^	0.21 ± 0.012 ^bA^	0.20 ± 0.007 ^bA^	0.13 ± 0.002 ^aB^
	16	0.16 ± 0.028 ^aA^	0.13 ± 0.035 ^aA^	0.16 ± 0.019 ^aA^	0.18 ± 0.021 ^bA^	0.10 ± 0.003 ^aB^

For each column (small letter) and for each row (capital letter), insignificantly differing values are labelled with the same letter (*p* > 0.05), and values labelled with a letter plus an asterisk indicate significant difference from each other, but no statistical difference from other equal letters. Data are shown as the mean ± SD, *n* = 4. Initial ion concentrations are presented in Table S1 (Supplementary Materials).

**Table 2:** Accumulation of pigments and total fatty acids in *Chlorella zofingiensis* cells exposed to the range of ABA concentrations in BBM medium.

Parameter	Day of Experiment	ABA, µM				
		0	1	5	10	50
Cell density, n × 10^7^/mL	16	0.831 ± 0.11 ^A^	1.251 ± 0.10 ^B^	1.319 ± 0.13 ^B^	1.539 ± 0.17 ^B^	2.343 ± 0.14 ^C^
Biomass, g/L dcw	16	0.71 ± 0.01^A^	1.09 ± 0.04 ^B^	1.18 ± 0.10 ^B^	1.28 ± 0.19 ^B^	2.37 ± 0.20 ^C^
TFA, pg/cell	3	9.44 ± 0.35 ^aA^	28.14 ± 2.425 ^aB^	12.94 ± 0.63 ^aC^	10.17 ± 1.02 ^aA^	45.58 ± 4.17 ^aD^
	7	11.14 ± 0.28 ^bA^	21.01 ± 0.65 ^bB^	9.68 ± 2.66 ^aC^	9.14 ± 1.32 ^aD^	13.86 ± 1.66 ^bF^
	16	11.21 ± 0.78 ^bA^	15.62 ± 1.15 ^cB^	8.83 ± 0.66 ^bC^	7.72 ± 0.41 ^bC^	17.69 ± 2.14 ^bD^
Production * TFA, mg/L	16	8.085 ± 0.58 ^A^	19.93 ± 1.33 ^B^	12.05 ± 0.90 ^C^	13.09 ± 0.97 ^C^	45.59 ± 3.77 ^D^
Chla+b, fg/cell	3	183.31 ± 12.08 ^aA^	341.64 ± 21.75 ^aB^	160.21 ± 1.52 ^aA^	165.38 ± 15.52 ^aA^	417.99 ± 67.81 ^aB^
	7	184.02 ± 16.70 ^aA^	409.72 ± 12.53 ^bB^	247.10 ± 42.54 ^bA^	171.96 ± 40.56 ^aA^	121.50 ± 7.26 ^bC^
	16	206.66 ± 10.77 ^aA^	368.87 ± 38.68 ^aB^	316.40 ± 20.85 ^cC^	265.35 ± 8.95 ^bD^	93.84 ± 23.55 ^bE^
Production * Chl, mg/L	16	1.72 ± 0.08 ^A^	4.61 ± 0.14 ^B^	2.78 ± 0.12 ^C^	1.96 ± 0.09 ^C^	2.28 ± 0.05 ^C^
CR, fg/cell	3	83.70 ± 8.38 ^aA^	133.06 ± 12.31 ^aB^	65.48 ± 8.85 ^aA^	63.62 ± 4.36 ^aC^	158.70 ± 13.87 ^aD^
	7	92.56 ± 8.05 ^aA^	132.96 ± 11.59 ^aB^	79.33 ± 15.87 ^aA^	69.32 ± 7.86 ^aC^	96.07 ± 11.21 ^bA^
	16	90.73 ± 9.07 ^aA^	107.13 ± 26.43 ^aA^	79.94 ± 15.99 ^aA^	45.36 ± 9.07 ^bB^	62.74 ± 12.55 ^cC^
Production * CR, mg/L	16	0.75 ± 0.007 ^A^	1.34 ± 0.12 ^B^	1.05 ± 0.06 ^C^	0.70 ± 0.02 ^A^	1.47 ± 0.15 ^B^
TFA/Chla+b	3	5.38 ± 1.20 ^aA^	8.41 ± 1.17 ^aB^	8.07 ± 0.89 ^aB^	6.10 ± 0.53 ^aA^	9.17 ± 0.91 ^aB^
	7	6.34 ± 1.88 ^aA^	5.17 ± 0.67 ^bA^	4.57 ± 0.57 ^bA^	6.50 ± 0.36 ^aA^	15.79 ± 2.63 ^bB^
	16	4.56 ± 0.55 ^aA^	3.89 ± 1.54 ^bA^	3.49 ± 0.28 ^cB^	5.12 ± 0.74 ^aA^	15.76 ± 1.19 ^bC^
TFA/CR	3	11.62 ± 2.18 ^aA^	23.06 ± 2.34 ^aB^	19.72 ± 1.65 ^aC^	15.94 ± 1.56 ^aA^	29.84 ± 4.18 ^aB^
	7	11.84 ± 1.69 ^aA^	15.07 ± 1.52 ^bA^	11.72 ± 1.28 ^bA^	17.32 ± 1.99 ^aB^	24.89 ± 2.23 ^aC^
	16	10.39 ± 1.02 ^aA^	12.70 ± 1.67 ^bA^	9.32 ± 1.21 ^bA^	12.18 ± 1.36 ^bA^	23.58 ± 1.11 ^bB^

TFA—total fatty acids; Chla+b—chlorophyll a + b; CR—carotenoids; dcw—dry cell weight. *—production on the day of test termination. For each column, values labelled with the same letter are not significantly different from each other (*p* > 0.05); for each row, values labelled with the same capital letter are not significantly different from each other (*p* > 0.05). Data are shown as the mean ± SD (for biomass and pigments *n* = 4; for cell density *n* = 12).

**Table 3:** Relationships between the exogenous ABA concentration and the abundance of the most represented bacteria in the microbiome of the *Chlorella zofingiensis* cultures exposed to a range of ABA concentrations in BBM medium for 16 days (the bacteria constituting 70% of the documented microbiome with |r| > 0.5 are shown).

Discovered Organism			ABA Added, µM					*r*
Order	Family	Genus	0	1	5	10	50	
Corynebacteriales	Nocardiaceae	*Rhodococcus*	36.5	50.7	40.77	41.81	69.96	0.90
Reyranellales	Reyranellaceae	*Reyranella*	19.7	17.2	14.52	11.81	9.66	−0.80
Rhizobiales	Xanthobacteraceae	*Bradyrhizobium*	9.80	8.95	8.43	8.16	4.43	−0.99

## Data Availability

Data are contained within the article and Supplementary Materials. Raw data were generated at the K.A. Timiryazev Institute of Plant Physiology, Russian Academy of Sciences and are available from the corresponding author [T.A.Kozlova] on request.

## Supplementary Materials

The following supporting information can be downloaded at: https://www.mdpi.com/article/10.3390/life13020452/s1, Table S1: Concentrations of compounds and ions in Bold’s Basal Medium (BBM), modified from Nichols and Bold [106]; Table S2: Correlation of the parameters reflecting growth and biochemical composition of the *Chlorella zofingiensis* cells exposed to the range of ABA concentrations in BBM medium for 16 days (see also Table 2).
