# Supplementary material for: Effect of Abscisic Acid on Growth, Fatty Acid Profile, and Pigment Composition of the Chlorophyte Chlorella (Chromochloris) zofingiensis and Its Co-Culture Microbiome
Source: Life (Basel). 2023 Feb 6;13(2):452. doi: 10.3390/life13020452 (PMC9962398; doi:10.3390/life13020452)
Supplement: Supplementary file 1 [file life-13-00452-s001.zip › life-2144935-supplementary.pdf]

Supplementary Material

Table S1. Concentrations of compounds and ions in Bold's Basal Medium (BBM), modified from Nichols and Bold [106]

Compounds composition			Ions composition	
Compound	Molar mass	Concentration, ppm	Ions	Concentration, ppm
KH_2PO_4	136.09	175	PO4	163.1
$CaCl_2 \times 2H_2O$/ Ca(NO3)$_2$ × $4H_2O$*	147.02/236.15*	25/45*	NO3	189.0
$MgSO_4 \times 7H_2O$	246.48	75	NH4	1.7
$NaNO_3$	84.99	250	SO4	31.97
K2HPO$_4$	174.20	75	Cl	31.1
NaCl	58.40	25	P	53.2
$Na_2EDTA \times 2H_2O$	372.24	10	N	7.4
$FeSO_4 \times 7H_2O$	278.02	5	Ca	11.8
H3BO$_3$	61.80	8	Mg	8.5
NH_4Cl	53.50	5	K	93.9
Trace Metals			Na	78.0
H_3BO_3	61.80	2.86	Fe	1.1
$MnCl_2 \times 4H_2O$	197.90	1.81	Cu	0.02
$ZnSO_4 \times 7H_2O$	246.47	0.22	Zn	0.23
Na2MoO$_4$ × $2H_2O$	241.90	0.39		
$CuSO_4 \times 5H_2O$	249.60	0.08		
$Co(NO_3)_2 \times 6H_2O$	291.00	0.049		

* compound used in the experiment

Table S2. Correlation of the parameters reflecting growth and biochemical composition of the *Chlorella zofingiensis* cells exposed to the range of ABA concentrations in BBM medium for 16 days (see also Table 2).

	ABA, μM	*Cell density, n x 10^7*	*Biomass, g/L*	*TFA, μg/cell*	*Ch b, ng/cell*	*CR, ng/cell*	*TFA/Chla+b*	*TFA/CR*
ABA, μM	1.00							
Cell density, n × 10^7	0.95	1.00						
Biomass, g/L	0.97	0.99	1.00					
TFA, μg/cell	0.60	0.51	0.58	1.00				
Chla + b, ng/cell	−0.81	−0.61	−0.66	−0.38	1.00			
CR, ng/cell	−0.50	−0.57	−0.49	0.35	0.48	1.00		
TFA/Chla + b	0.98	0.89	0.93	0.67	−0.86	−0.41	1.00	
TFA/CR	0.96	0.90	0.93	0.77	−0.75	−0.33	0.98	1.00

Chlorophyll-a and -b (Chla + b), total carotenoid (CR), and total fatty acid (TFA)

Reference:

1. Nichols, H.W.; Bold, H.C. *Trichosarcina polymorpha* gen. et Sp. nov. *J. Phycol.* **1965**, *1*, 34–38.
